# A prospective study of the impact of an emotional intelligence program on opioid relapse and its determinants in upper Egypt

**DOI:** 10.1186/s40359-025-02638-8

**Published:** 2025-04-10

**Authors:** Hanan Faried Maghawry, Alaa M. Darwish, Naglaa Abd Elmeged Mohammed, Nadia Abd El-ghany Abd El-hameed, Gellan K. Ahmed

**Affiliations:** 1 Department of Psychiatric and Mental Health Nursing, Faculty of Nursing, Assiut University, Assiut, Egypt; 2 Department of Neurology and Psychiatry, Assiut University, Assiut, 71516 Egypt

**Keywords:** Opioid, Emotional intelligence, Psychiatric comorbidities, Relapse, Addiction

## Abstract

**Background:**

Opioid use is a growing concern worldwide with high relapse rates and psychiatric comorbidities. Emotional intelligence (EI) has been associated with positive health outcomes, yet limited research exists on EI-based interventions for opioid use disorders. The study aimed to assess EI levels among individuals with opioid addiction and implement an EI program to reduce addiction severity and relapse rates, as well as examine potential factors that contribute to enhancing EI.

**Methods:**

A quasi-experimental study assigned 40 males with opioid use disorder into an intervention group receiving a 2-week EI program (*n* = 20) and a control group (*n* = 20). Healthy comparison group (without opioid use disorder) (*n* = 20) was also included. Measures including the Emotional Intelligence Scale, Personality Inventory for DSM-5, Symptom Checklist-90-Revised (SCL-90-R), Addiction Severity Index, and Advance Warning of Relapse (AWARE) questionnaire were used to assess the groups at baseline,2 weeks and 3-month follow-up in patients with opioid use disorder.

**Results:**

The healthy group exhibited lower scores on disinhibited personality subscales, maladaptive personality traits, and SCL-90-R subscales than the opioid addiction groups at baseline. The EI intervention group displayed substantial increases in EI subscale scores at the second and third follow-up assessments compared to the control group. At AWARE scale follow-up, all control group patients had relapsed, versus only one patient in the EI intervention group.

**Conclusions:**

Participants with opioid addiction demonstrated lower baseline EI and higher rates of psychiatric symptoms and maladaptive personality traits than healthy group. A brief EI intervention led to significant EI increases and lower relapse rate versus standard treatment alone.

## Background

There is a growing prevalence of substance addiction in Egypt, accompanied by changes in substance use patterns. Opiates have become the most utilized substance [1]. Opioid use addiction relapse rates outnumber those of other substance addictions [2]. Over the last decade, the number of drug overdose deaths has risen dramatically due to an increase in prescription opioid-related overdoses [3]. This trend has been associated with high rates of overdose deaths and a rise in the incidence of diseases related to intravenous drug use, such as the human immunodeficiency viruses (HIV) and Hepatitis C [4]. Individuals suffering from opioid addiction have a mortality risk that is more than four times higher than the general population, resulting in a life expectancy loss of more than nine years compared to the general population [5].

Several factors are linked to substance addiction. Genetics, pharmaceutical effects, peer pressure, emotional stress, anxiety, depression, and environmental stress are all possible contributors [6]. Opioids have a biological basis for addiction since they lack neurotransmitters like dopamine, boosting the desire to seek out external sources of endorphins. Some people may turn to opioids to make up for this shortfall. Peer pressure or a past physician’s prescription for an injury can have an environmental influence on opiate use [7].

Martinotti et al. claim that there is a direct link between substance addiction and psychological effects such as mood swings, anger, despair, anxiety, and even suicidal ideation. These effects are not only temporary, but they also last throughout drug use. Substance addiction, particularly opioid addiction, is linked to decreased concentration, forgetfulness, and increased paranoid severity [8]. Individuals addicted to opioids frequently have a high frequency of mental comorbidities such as depression, anxiety, and personality disorders, as well as an stimulant- or drug-induced psychoses, substance-induced mood disorders, and substance-induced anxiety problems [9]. Furthermore, those with substance addiction who have psychiatric disorders face diagnostic and therapeutic challenges as they have a poor recovery prognosis and more severe psychosocial damage [10]. In addition, those who are addicted to substances share personality traits such as borderline personality, anxiety, despair, tension, and different negative feelings. Specific personality features are more likely to lead to the development of substance addiction. Identifying disorders linked to substance addiction also prove the importance of normal and abnormal personality traits [11].

Emotions play a role in the development of psychotropic substance addiction.It has been reported that emotions such as pain, disappointment, or a desire to escape an awful circumstance can develop substance addiction [12]. Emotional intelligence is a concept that improves people’s emotional awareness, allowing them to spend less time problem-solving and feel more in control of their lives [13]. Emotional intelligence (EI) is defined as the ability to recognise and control one’s own emotions as well as the emotions of others in order to enhance personal and interpersonal emotional and intellectual growth [14]. EI components, such as the ability to identify and use emotions effectively, as well as the overall emotional intelligence score, are strongly associated with substance addiction [15, 16]. In this regard, Hosseini and Anari reported emotional intelligence as connected with overall health, optimism, stress management, marital satisfaction, social compatibility, anger control, and creative problem-solving [17].

Poor emotional intelligence on the other hand, is linked to violent behaviour, despair, addiction, and criminality, as these concerns reflect an individual’s incapacity to handle emotions [16]. Emotional intelligence is critical in preventing drug relapse and promoting mental health [18]. Individuals with high emotional intelligence tend to have better health outcomes and manage their lives more successfully by modifying their goals and objectives in response to internal and external reality. Furthermore, they are more likely to maintain positive traits like autonomy, self-acceptance, positive relationships, a sense of purpose, and personal development, all of which are protective against mental illnesses [19]. In the context of substance addiction, two critical components of emotional intelligence are that “decoding and differentiation of emotions” and “regulation of emotions” [20].

Researchers have continually attempted to understand the basic causes of substance addiction as well as potential therapies. While emotional management has been identified as a crucial element, the impact of emotional intelligence on this issue has not been completely evaluated [16]. Therefore, this study aims to assess the levels of emotional intelligence among opioid addiction and implement an emotional intelligence program to evaluate its effects on reducing addiction severity and relapse rates. Additionally, to evaluate the relevant parameters that enhance emotional intelligence.

According to the Diagnostic and Statistical Manual of Mental Disorders, Fifth Edition (DSM-5) [21]. Substance addiction is a chronic, recurring disorder characterised by compulsive drug seeking, continuing use despite negative effects, and long-term brain alterations. It is considered both a mental illness and a complex brain disorder. Ssubstance addiction is a medical condition resulting from persistent substance abuse [22]. Addiction relapse is defined as the reappearance of behavioural symptoms or other significant signs of active disease following a period of remission [23].

## Methods

### Design

The research employed a quantitative approach with a quasi-experimental design known as a pre-post-test control group design.

### Sample and sample size

The sample size was determined using the EPI Info statistical program version 7, based on a previous study conducted by A Santosh [24]. Using parameters of 0.5 proportion, 95% confidence level, and 5% margin of error, calculations indicated a minimum requirement of 20 participants each for the EI group, control group and healthy group.

The healthy group comprised males with no history of substance addiction, neurological or medical issues, or psychiatric disorders. Due to the withdrawal of one participant from EI group, the final sample size was 39.

For group assignment between the EI and control groups, simple randomization was implemented using admission ticket numbers: participants with even numbers were assigned to the EI group, while those with odd numbers went to the control group. Study eligibility criteria required participants in the EI and control groups to be male, at least 18 years old, and meet DSM-5 criteria for opioid addiction. Exclusion criteria ruled out individuals with cognitive impairment, neurological conditions, or medical illnesses.

### Procedure

Between January 2022 and November 2022, a total of 40 opioid patients were recruited. The participants were divided into two groups: those with opioid addiction undertaking EI program, and individuals with opioid addiction who did not get EI program. The study was conducted in the Addiction Management Unit inpatient sector and outpatient clinic at the Psychiatry, Neurology, and Neurosurgery Hospital of Assiut University, at Assiut city in Egypt. Patients with opioid use disorder were assessed at baseline, 2 weeks, and a 3-month follow-up, while the healthy group was assessed once.

### Pilot study

A pilot study was conducted with a group of five patients (not included in the study population) to evaluate the objectivity and clarity of the tools.

### Study tools

After obtaining written informed consent, the following tests were administered to all patients:

### Psychiatric interview

Comprehensive psychological and medical evaluations were conducted before the start of the trial. A semi-structured form was developed to screen sociodemographic and clinical characteristics and also gather medical histories. The collected data included age, gender, level of education, occupation, marital status, and clinical information such as diagnosis, substance use amount, frequency, duration, and motivations for initiation.

### Family socioeconomic status scale

This scale, developed by Sawsan and AF [25] and updated by El-Gilany, et al. [26] consists of 7 domains: education and culture, occupation, family, family possessions, economics, home sanitation, and healthcare access. The total score of the scale is 84, with scores below 42 indicating a very low level of socioeconomic status, scores between 42 and less than 63 indicating a low level of socioeconomic status, scores between 63 and less than 71.4 indicating a middle level of socioeconomic status, and scores between 71.4 and 84 indicating a high level of socioeconomic status.

### Emotional intelligence scale

The Emotional Intelligence Scale (EIS) was originally developed by Bar-On as a measurement tool for assessing emotional intelligence. It comprises 60 items categorized into six domains: Personal competence, social competence, stress management, adaptation, general mood, and positive impression [27]. The scale utilizes a four-point Likert scale ranging from 1 (never) to 4 (always). The scores are converted into a percent score, with emotional intelligence considered high if the percent score is above 75%, moderate if it falls between 60% and 75%, and low if it is below 60%. To assess the scale’s internal consistency, Cronbach’s Alpha coefficient was computed and yielded a value of 0.910. The correlations among the different domains of emotional intelligence and the patterns of convergent and discriminant validities indicated that the scale encompasses a broad range of emotional constructs.

### Personality inventory for DSM-5 (PID-5)

The Personality Inventory for DSM-5—Brief Form (PID-5-BF)—Adult is a 25-item self-rated assessment scale designed to evaluate personality traits in individuals aged 18 and older [28]. It measures five domains of personality traits, including negative affect, detachment, antagonism, disinhibition, and psychoticism, with each domain consisting of five items. Each item is rated on a 4-point scale. The overall score on the scale ranges from 0 to 75, with higher scores indicating a greater overall presence of personality disorder. The scores for each personality trait domain range from 0 to 15, with higher scores indicating a higher level of dysfunction in that specific trait category. To assess the reliability of the inventory, internal consistency was determined using Cronbach’s alpha values, which ranged from 0.83 to 0.89, and test-retest coefficients, which ranged from 0.77 to 0.87 [29]. A cutoff score of ≥ 2 was used for each of the five subdomains score, based on previous research [30].

### The symptom Checklist-90–Revised (SCL-90-R)

The Symptom Checklist-90–Revised (SCL-90-R) is a 90-item self-report symptom scale developed by Clinical Psychometric Research to assess the psychological symptom patterns of psychiatric and medical patients [31]. The revised (R) form of the scale was subsequently modified and validated by Lr [32]. While an Arabic version of the original SCL-90-R was earlier available, the validation and standardization of the Arabic translation were recently completed by Elbehairy [33]. Each item on the scale is assessed on a distress scale ranging from 1 to 5, with responses varying from “none” to “extremely.” The scale consists of nine primary symptom dimensions: somatization, obsessive-compulsive, interpersonal sensitivity, depression, anxiety, hostility, phobic anxiety, paranoid ideation, and psychoticism. For this study, the researchers focused on the 47-item symptom dimensions of obsessive-compulsive, depression, anxiety, phobic anxiety, and paranoid ideation. The scale demonstrated good reliability with a Cronbach’s Alpha score of 0.90.

### The addiction severity index (ASI)

It was developed by McLellan, et al. as an interview-based instrument to assess the history, frequency, and consequences of alcohol and drug use [34]. The ASI provides a comprehensive overview of substance addiction across multiple domains, including medical status, employment and support, drug use, alcohol use, legal status, family and social status, and psychiatric status. The scale consists of 200 questions divided into seven subscales. Scoring on the ASI ranges from 0 to 9, with higher scores indicating more severe problems. Internal consistency reliability of the ASI was examined using Cronbach’s alpha coefficients, which ranged from 0.64 to 0.77 across different domains. Test-retest reliability and concurrent validity were also assessed and showed moderate to high levels of reliability and validity for the ASI composite scores [35].

### The advance warning of relapse (AWARE) questionnaire

It was developed to measure relapse warning signs [36]. The questionnaire was refined from its original 37-item version to the current 28-item scale (version 3.0) by Miller et al. [37]. A panel of expert medical psychiatrists and psychiatric nursing professors reviewed the questionnaire’s Arabic translation. The scale has a score range of 28 to 196, with higher scores indicating a greater likelihood of relapse. Internal consistency reliability was assessed using Cronbach’s alpha, which yielded a coefficient of 0.962, indicating extremely high internal consistency. Confirmatory factor analysis confirmed that the questionnaire measures a single factor. Convergent validity was established by comparing the AWARE Questionnaire with the Brief Symptom Inventory-18, a well-known mental distress measure predicting relapse [38].

### The emotional intelligence program

#### Overview of the emotional intelligence program

Sessions were held for two weeks, three times a week, and with each session lasting more than 1 h. There were seven sessions, including six instructional sessions and one pre-assessment (first interview) session. The sessions incorporated lectures, videos, and group discussions.

#### Phases of the educational program

The study researchers developed the program after four months of evaluating relevant materials and an emotional intelligence program.

#### Assessment phase

This phase involved assessing the emotional intelligence levels of individual with opioid addiction. Stable, non-aggressive, and cooperative opioid addiction were interviewed to gather sociodemographic data, clinical data, family socioeconomic status, personality characteristics, psychotic symptoms, addiction severity index, and emotional intelligence levels (first interview followed by six emotional intelligence sessions for EI group) and (first interview only for control group ). Group 3 were assessed for demographic characteristics, family socioeconomic status, and emotional intelligence.

#### Considerations of the program’s content

The program was developed based on literature describing emotional intelligence components and their relevance to patients with substance addiction. The content was revised by experts, including a medical psychiatrist from the faculty of medicine and two professors of psychiatric nursing from the faculty of nursing at Assiut University.

#### Content of the program

- Session 1: Definition of emotional intelligence, the importance of emotional intelligence, and components of emotional intelligence.
- Session 2: Self-awareness and independence.
- Session 3: Empathy and social relationships.
- Session 4: Social responsibility.
- Session 5: Tolerating stress.
- Session 6: Impulse control and problem-solving.

#### Putting the program into action

In this phase, the program’s strategy, including the time, number of sessions, and teaching methods, was implemented for EI group. The suitability of the teaching location and facilities was evaluated. The sessions occurred in the recreation hall, where the content was presented, emotional intelligence videos were displayed on a television screen, and patients received a pre-designed booklet containing the program’s content. The outcomes of each session were reviewed in the following session to ensure understanding.

#### Evaluation of the program’s impact

The emotional intelligence levels of the patients were evaluated at a post-test for EI group after completing the program and for control group before discharge from the hospital. Follow-up evaluations were conducted after three months for both groups, using emotional intelligence assessments, the addiction severity index, and the advance warning of relapse. The evaluation was conducted through a direct interview at the outpatient clinic or via telephone for patients who were unable to attend the clinic or were traveling away.

### Statistical analysis

Data entry and statistical analysis were performed using the SPSS 26 Statistical Software Package. Descriptive statistics were used for qualitative data, including numbers and percentages. The χ2 test or Fisher’s exact test was used to compare between groups for categorical variables. Quantitative data were described using mean and standard deviation (mean ± SD) or median (interquartile range). The Kruskal-Wallis’s test was used for non-normally distributed data among more than two groups, and the Mann-Whitney U test was used to compare two groups. The Friedman test was used to compare means across three related groups.

To address multicollinearity among the Addiction Severity Index subscales, we conducted a correlation analysis using Spearman’s correlation. Variables with correlation coefficients *r* > 0.7 were considered to exhibit strong multicollinearity. Based on this analysis, we found strong correlations between Employment status and Psychiatric status (*r* = 0.772), as well as between Psychiatric status and Family and social status (*r* = 0.759). To reduce redundancy while maintaining key indicators of addiction severity, we selected Drug abuse and Psychiatric status subscales for inclusion in the regression model. These variables were chosen based on their relevance to addiction treatment outcomes and their ability to capture broader aspects of addiction severity, while minimizing multicollinearity in the model.

Multivariate regression analysis was employed to explore the relationship between the change in total emotional intelligence (after the program measured before the program and at the 3-month follow-up) and other variables. Five dependent variables were included in the regression model, which would ideally require a sample size of 50 participants. However, due to study constraints, our sample was limited to 40 participants, a limitation that has been addressed in the discussion section. Statistical significance was defined as a p-value of less than 0.05.

## Results

### Sociodemographic characteristics

In this study, three groups of participants were enrolled: EI group consisted of individuals with opioid addiction who received EI program, control group consisted of individuals with opioid addiction without EI program, and group 3 consisted of a healthy participant. There were no significant statistical differences in sociodemographic characteristics between the opioid addiction group and the healthy group. Most participants were married manual workers, below 30 years of age, had a secondary level of education, lived in urban areas, and belonged to a low socioeconomic status (See Table 1).

### Clinical characteristics of opioid addiction

Table 2 presents significant statistical differences between the two opioid addiction groups in terms of the duration of opioid use, EI group had a higher mean duration of opioid use compared to control group. Both groups predominantly used inhalation methods, and motivation for opioid use was associated with negative peer influences.

### PID-5 and SCL-90- R

Table 3 demonstrates significant statistical differences among the three groups regarding disinhibition and the total scores of the personality traits scale. The healthy group had a lower frequency (45%) and the mean score of disinhibitions (3.15 ± 1.30) compared to the other groups. The total score of the personality traits scale was lower in the healthy group (20.90 ± 4.75) compared to the other groups. (See Table 3)

Regarding the SCL-90-R, there were no significant statistical differences among the three groups. The subscales of the SCL-90-R showed lower frequencies in the healthy group compared to the other groups. (See Table 4)

### Emotional intelligence

Baseline assessment of emotional intelligence revealed that the mean scores of EI subscales were lower in the case groups compared to the healthy group, with significant differences between the groups. Follow-up assessments at the second and third-time points showed a gradual increase in EI subscale scores in EI group compared to control group, with significant differences. In contrast, control group showed an increase in mean EI subscale scores at the second follow-up compared to the baseline scores of the same group. However, there were almost no changes in the third follow-up compared to the baseline measure in the same group, with no significant statistical differences among the measures within the group. (See Table 5)

### Addiction severity index (ASI)

A comparison within each group revealed significant statistical differences between the two time points (baseline and 3rd follow-up) regarding employment, drug abuse, family and social status, and psychiatric status. EI group showed improvement in severe problems within these subscales, with a reduction in the percentage at the 3rd follow-up compared to the baseline of the same group. On the other hand, control group experienced worsening severe problems within these subscales, with an increase in frequency at the 3rd follow-up compared to the baseline of the same group. (See Table 6)

At baseline assessment, there were significant statistical differences among both groups in ASI scores regarding employment, drug abuse, family and social status, and psychiatric status. In terms of these subscales at baseline, EI group had a higher frequency of severe problems than control group. In contrast, control group had a higher percentage of mild to moderate problems than EI group. Furthermore, at the 3rd follow-up assessment, EI group demonstrated improvements in the frequency of severe problems in drug abuse and family and social status compared to control group.

### Advance warning of relapse

**Fig. 1:**
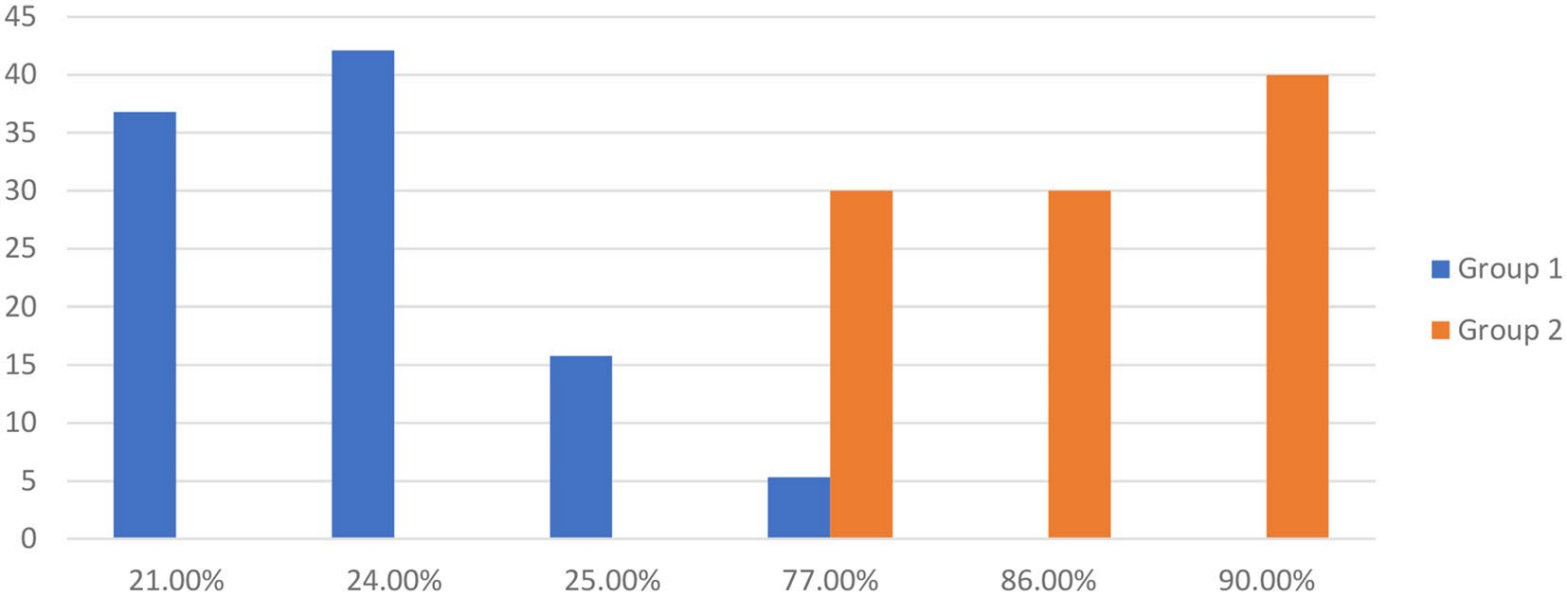
AWARE scores among cases groups at 3rd follow-up measure

Significant statistical differences were observed between the two groups in the follow-up evaluation. Individuals with opioid addiction who did not receive the emotional intelligence program had a higher mean score (123.85 ± 16.55) than the other group (76.53 ± 14.44). Additionally, all individuals in control group experienced relapse, while only one individual in EI group had relapse (See the Fig. 1).

### Regression

In the multivariate regression model, only participation in the emotional intelligence program remained a significant predictor of emotional intelligence improvement (See Table 7).

## Discussion

Relapse is a complicated behavioural and psychological problem defined by individuals in addiction recovery’s failure to effectively control their desire to use drugs [39]. This challenge is closely linked to emotional intelligence (EI) and coping mechanisms. For instance, individuals who struggle with stress management and have heightened emotional responses often turn to opiates as a maladaptive coping strategy [16].

Emotional intelligence plays a crucial role in addiction recovery and relapse prevention. Research indicates that individuals with higher emotional intelligence are less prone to relapse compared to those with lower emotional intelligence [40]. This relationship can be explained by several factors related to emotional awareness, regulation, and coping abilities.

Individuals with low emotional awareness, limited knowledge about appropriate emotional expression, and poor mood control may struggle in various areas of life, particularly stress management. Maladaptive coping methods may serve as a link between low EI and substance use disorders. Data suggests that these ineffective strategies are associated with the initiation, maintenance, and relapse stages of substance use [41].

The way individuals respond to stress is also influenced by their level of emotional intelligence. Those with high emotional intelligence can regulate their feelings and emotions in high-pressure situations, while individuals lacking emotional intelligence struggle to control their emotions when faced with life’s pressures. This connection between low emotional intelligence and addiction can be understood as a response to stress [42].

In high-stress situations, individuals with inadequate emotional intelligence may turn to substance use as a means of coping, increasing their vulnerability to addiction and relapse.Therefore, this study aimed to assess the levels of emotional intelligence among individuals with opioid addiction, implement an emotional intelligence program, and evaluate its impact on opioid relapse. Additionally, the study aimed to identify relevant factors that may enhance emotional intelligence.

Personality traits play a crucial role in understanding substance addiction and related behaviours. Regarding the personality scale, the healthy group exhibited lower mean scores and frequencies on the disinhibited subscale and the total score of the personality traits scale compared to individuals with opioid addiction. Notably, at least one of the DSM-5 criteria for substance use disorders specifies an impaired ability to control behaviour, indicating that disinhibition is a defining characteristic of addiction [43].

Personality traits are considered a primary aspect of individual variation and a crucial element in enhancing patients’ understanding and the context in which addictive behaviors arise. Temperament dimensions, as essential biological components of personality, have a significant impact on the manifestation of addictive behaviors. Therefore, personality traits are primary predictors of substance addiction behaviors [44].

The manifestation of drug-using behaviors is associated with highly impulsive personality traits and emotional instability. Furthermore, substance addiction is frequently linked to compromised response inhibition [45].

There is a plausible explanation that chronic excessive substance use may increase disinhibition through neurotoxic effects on the prefrontal cortex [46, 47]. These prefrontal regions are implicated in executive functions, including inhibitory control. Thus, structural and functional injury to these regions can result in disinhibited behaviour [48–50]. This neurobiological perspective provides insight into the relationship between substance use, brain function, and the expression of personality traits associated with addiction.

Moreover, most individuals with opioid addiction demonstrated personality traits associated with negative affect, detachment, antagonism, disinhibition, and psychoticism in this study. These traits were also present, albeit to a lesser extent, in healthy individuals. The heightened presence of these traits in individuals with substance addiction can be attributed to the complex interplay between personality and addiction mechanisms.

Substance addiction is often driven by a need to manage unpleasant emotional experiences, which paradoxically intensifies negative affectivity and discourages individuals from seeking alternative forms of relief. This negative affectivity, an internalizing personality trait, frequently manifests as depressive, anxious, withdrawn, and anhedonia emotional states. Detachment, another significant trait, is associated with beliefs indicating a lack of interest in relationships, mistrust of others, and interpersonal ambivalence. Antagonistic characteristics, on the other hand, are linked to an exaggerated sense of superiority, attitudes enabling hostile behaviors towards others, and concerns about being controlled by others. Previous research has shown that when comparing individuals with substance use disorders to the general population, medium effect sizes were observed in three personality domains: Disinhibition, Psychoticism, and Antagonism [51]. Consistent with this, a previous study found that compared to non-users, drug users demonstrated higher performance on tests measuring negative affectivity, antagonism, disinhibition, and psychoticism. This study highlighted the differences between pathological and normal personality models in drug users and non-users. Moreover, antagonism and disinhibition are recognized as externalized pathological traits characterized by grandiosity, impulsivity, risk-taking, and reckless behaviour. The antisocial and borderline traits, identified as determining factors in substance addiction, also overlap with these dimensions [11].

It is important to note, however, that there remains limited knowledge regarding the predictive role of detachment and hostility in human emotional reactions to interpersonal pressures encountered in everyday life [52]. This gap in understanding highlights the need for further research to elucidate the complex relationships between personality traits, emotional responses, and substance addiction.

Regarding comorbid psychiatric conditions, the healthy group had a lower percentage of abnormal response in all subscales SCL-90-R compared to other groups. In contrast, individuals with opioid addiction in both groups had higher frequency abnormal responses in obsessive-compulsive, depression, phobic anxiety, and paranoid ideation than the healthy group. This finding suggests that individuals with depression may turn to opioid use to alleviate negative moods, and the subsequent improvement in their mood state increases the reinforcing value of drug use. These findings align with McBrain’s study, which identified self-regard as a primary predictor of depression, followed by problem-solving ability as a secondary predictor. Depression is characterized by indecision and problem-solving difficulties. Depressed individuals maintain negative perspectives about themselves, others, and the future. Their reluctance to make decisions stems from both fear of failure and depression-related symptoms such as apathy and motivational deficits, which impair their problem-solving capabilities [53].

Previous studies found that a majority of substance use addicts exhibited comorbid depression, followed by suicidality, generalized anxiety disorder, panic disorder, specific phobia, social phobia, schizophrenia, and bipolar disorder without personality problems [2].

According to EI, this study demonstrated a significant improvement in emotional intelligence domains among individuals with opioid addiction after two weeks and three months of receiving the emotional intelligence program compared to group who did not receive it.

A previous study implemented an educational program based on the ability model of emotional intelligence to enhance emotional intelligence among adolescents. The study showed that emotional intelligence training produced beneficial and long-lasting changes in emotional performance and helped control behaviour [54]. Previous study has shown that enhancing EI abilities has a beneficial effect on coping mechanisms and problem-solving capabilities, thereby decreasing negative behaviours and harmful stress management strategies like substance use [53].

Enhancing and strengthening EI enables individuals to enhance their capacity to control their emotional manifestations, regulate their emotions, and have a deeper understanding of their influence on their peers, family, and communities [53]. Therefore, teaching individuals how to manage their emotions can be valuable for behaviour control and improving their ability to resist substance addiction.

Regarding an addiction severity index, individuals with opioid addiction who received the EI program experienced improvements in various areas, including employment, drug abuse, family and social status, and psychiatric status, during the third follow-up compared to the baseline. In contrast, patients who did not receive the EI program showed an increase in severe problems on these subscales, as evidenced by a higher frequency in the third follow-up. This can be attributed to the reduced relapse rates in patients who received the emotional intelligence program, leading to a decrease in the severity of addiction-related issues compared to those who did not receive.

A previous study found that baseline addiction severity significantly predicted both psychiatric and medical status domains, indicating that individuals with higher baseline addiction severity exhibited more significant addiction-related psychiatric and medical problems, respectively [55].

The research revealed that patients actively using drugs exhibited severity levels equal to or greater than those who were abstinent. Significant statistical differences were found between these two patient groups across multiple domains: legal, mental health, drug use, and alcohol use [56]. Furthermore, previous studies have demonstrated that heroin use is associated with high relapse risk, emphasizing the importance of maintaining strong participant retention in treatment programs, though there was evidence of decreased addiction severity over time [57]. So, improving the addiction severity scale emphasizes improving addiction-related psychiatric and medical issues.

Regarding relapse, our study found out that that individuals with opioid addiction who participated in the emotional intelligence program had a reduced relapse rate than those who did not. This implies that those with higher emotional intelligence are more capable of anticipating others’ demands, interpreting peer pressure, controlling their emotions, and declining substance usage offers. Similarly, Davis noted an association between better emotional intelligence and fewer relapses in the first six months after quitting substance use and smoking [58]. Furthermore, Du et al. observed that people who can control and manage their emotions are more likely to use appropriate coping methods, especially when confronted with difficult situations involving substance offers [59]. Another study by Nawi et al. found that difficulties with emotion control might lead to an addiction tendency [60].

Changes in total emotional intelligence (before and after three months of follow-up) and program participation and psychometric scales were investigated using a multivariate regression model. Emotional intelligence improved more in individuals with opioid addictions who got the emotional intelligence program, this could explained that EI program was contained value information and introduced in a continued way from assessment to the follow-up duration this allowed to increase their EI level.

### Limitations and recommendations

The first limitation of this study was a small sample size, which may have impacted the findings’ generalizability. Furthermore, the researchers experienced challenges in managing the behaviours of individuals with substance addiction, which resulted in more time spent with them. Furthermore, collecting relapse outcome information solely through the AWARE scale may have overlooked other factors.

Based on the results of this study, it is recommended to implement emotional intelligence programs as part of the treatment for patients with substance addiction. These programs should equip patients with the necessary skills to cope effectively with situations that may trigger substance reuse in their daily lives. Educational courses can be utilized to enhance emotional intelligence to prevent opioid addiction before it occurs. Further research is needed to assess emotional intelligence levels and their effects across different types of substance use disorders and behavioral addictions.

## Conclusions

In conclusion, this study demonstrated that individuals with opioid addiction exhibit low levels of emotional intelligence and experience a higher prevalence of comorbid psychiatric symptoms and personality traits than healthy individuals. The findings highlight the positive impact of emotional intelligence on preventing opioid relapse. These results can inform psychiatrists and addiction counsellors in developing more effective approaches to aid individuals in quitting drug use. Additionally, the findings can be applied to primary and secondary substance addiction prevention strategies.

**Table 1:** Socio-demographic characteristics of the studied groups (cases **and healthy control)**

Socio-demographic characteristics	Cases group *n* = 39		Healthy control group *n* = 20		Chi or U value	*P*-value
	No.	%	No.	%		
**Age**: (Mean ± SD) Median (interquartile Range)	29.85 ± 7.45 28 (9)		30.15 ± 9.10 25 (12.5)		372.5	0.779
< 30	22	56.4	12	60.0	1.042	0.594
30–40	12	30.8	4	20.0		
> 40	5	12.8	4	20.0		
**Residence**:					1.846	0.174
Rural	9	23.1	8	40.0		
Urban	30	76.9	12	60.0		
**Levels of education**:					4.330	0.228
Read and write	3	7.7	2	10.0		
Preparatory	6	15.4	4	20.0		
Secondary	24	61.5	7	35.0		
University	6	15.4	7	35.0		
**Marital status**:					0.234	0.628
Single	15	38.5	9	45.0		
Married	24	61.5	11	55.0		
**Occupation**:					0.406	0.524
Manual workers	32	82.1	15	75.0		
Employees	7	17.9	5	25.0		
**Family socioeconomic status**					2.669	0.263
Very low	16	41.0	4	20.0		
Low	18	46.2	12	60.0		
Middle	5	12.8	4	20.0		

Chi square test U: Mann Whitney U test

**Table 2:** Clinical characteristics of cases groups

Clinical characteristics	Group 1 *n* = 19		Group 2 *n* = 20		Chi or U value	*P*-value
	No.	%	No.	%		
^**#**^**Methods of drug use**:					7.104	0.069
Oral	8	26.7	4	16.7		
Inhalation	15	50.0	18	75.0		
Injection	7	23.3	2	8.3		
**Age of starting abuse (in years)**: (Mean ± SD) Median (interquartile Range)	22.00 ± 7.53 20 (10)		23.55 ± 5.30 24 (7.75)		148.0	0.236
< 20	9	47.4	6	30.0	1.464	0.481
20–30	7	36.8	11	55.0		
> 30	3	15.8	3	15.0		
**Duration of abuse (days) (Mean ± SD)** **Median (interquartile Range)**	3306.95 ± 2681.61 3360(4704)		1478.40 ± 2216.47 672(1008)		102.500	0.013*
^**#**^**Motivation for initiation**:					5.295	0.381
Bad friends	14	53.8	18	56.3		
Trial	6	23.2	10	31.2		
Increase strength and energy	1	3.8	0	0.0		
Escape from life stressors	4	15.4	4	12.5		
Relieve chronic pain	1	3.8	0	0.0		

Chi square test U: Mann Whitney U test #More than answer * Statistically significant difference (*p* < 0.05)

Group1: opioid addicts who have emotion intelligence program, group 2: opioid addicts who did not have emotion intelligence program

**Table 3:** Total of personality inventory subscales of the studied groups

Personality inventory subscales	Group 1 *n* = 19	Group 2 *n* = 20	Group 3 *n* = 20	Chi /k value	*P*-value
Negative affect (mean ± SD) Median (interquartile Range)	4.736 ± 1.52 5(2)	4.60 ± 1.87 4.5(2.75)	4.05 ± 1.46 4(5)	2.106	0.349
Normal	4(21.1%)	5(25%)	5(25%)	0.111	0.946
Abnormal	15(78.9%)	15(75%)	15(75%)		
Detachment(mean ± SD) Median (interquartile Range)	5.421 ± 2 4(3)	4.85 ± 1.56 5(1.75)	4.20 ± 1.54 4(1.75)	3.058	0.217
Normal	2(10.5%)	2(10%)	5(25%)	2.225	0.329
Abnormal	17(89.5%)	18(90%)	15(75%)		
Antagonism(mean ± SD) Median (interquartile Range)	6.10 ± 1.24 6(2)	5.30 ± 2.15 5.50(3)	4.85 ± 1.89 5(2)	4.048	0.132
Normal	0(0%)	4(20%)	4(20%)	4.396	0.111
Abnormal	19(100%)	16(80%)	16(80%)		
Disinhibition(mean ± SD) Median (interquartile Range)	4.31 ± 1.33 4(0)	3.65 ± 1.18 4(1)	3.15 ± 1.30 3(2)	7.883	0.019*
Normal	2(10.5%)	6(30%)	11(55%)	8.894	0.012*
Abnormal	17(89.5%)	14(70%)	9(45%)		
Psychoticism(mean ± SD) Median (interquartile Range)	5.31 ± 1.24 5(1)	5 ± 1.33 5(2)	4.85 ± 1.42 4.5(2)	1.697	0.428
Normal	2(10.5%)	2(10%)	3(15%)	0.278	0.866
Abnormal	17(89.5%)	18(90%)	17(85%)		
Total PID (mean ± SD) Median (interquartile Range)	26.26 ± 3.61 25(5)	23.4 ± 4.88 23(4.75)	20.90 ± 4.75 20.5(7.50)	11.874	0.003*

Chi square test K: The Kruskal-Wallis test * Statistically significant difference (*p* < 0.05)

Group1: opioid addicts who have emotion intelligence program, group 2: opioid addicts who did not have emotion intelligence program, group 3: Healthy control group, PID: Personality inventory for DSM-5

**Table 4:** Distribution of symptoms checklist and its subscales among studied groups

Variables	Group 1 *n* = 19	Group 2 *n* = 20	Group 3 *n* = 20	Chi value	*P*-value
Obsessive compulsive					
Normal	9(47.4%)	10(50%)	16(80%%)	5.389	0.068
Abnormal	10(52.6%)	10(50%)	4(20%)		
**Depression**					
Normal	9(47.4%)	13(65%)	16(80%%)	4.530	0.104
Abnormal	10(52.6%)	7(35%)	4(20%)		
**Anxiety**					
Normal	10(52.6%)	10(50%)	10(50%)	0.036	0.982
Abnormal	9(47.4%)	10(50%)	10(50%)		
**Phobic anxiety**					
Normal	3(15.8%)	4(20%)	5(25%)	0.512	0.774
Abnormal	16(84.2%)	16(80%)	15(75%)		
**Paranoid ideation**					
Normal	5(26.3%)	10(50%)	11(55%)	3.685	0.158
Abnormal	14(73.7%)	10(50%)	9(45%)		

Chi square test * Statistically significant difference (*p* < 0.05)

Group1: opioid addicts who have emotion intelligence program, group 2: opioid addicts who did not have emotion intelligence program, group 3: Healthy control group

**Table 5:** Emotional intelligence among the studied groups

Groups	Baseline time (T1)	2 nd follow-up time (T2)	3rd follow-up time (T3)	*p*-value of 3 measures in the same group	*p*-value of baseline time of 3 groups	*p*-value of 2nd follow-up time of 2 groups	*p*-value of 3rd follow-up time of 2 groups	The Friedman test time X group interaction
Personal competence								
**Group 1**	9.32 ± 2.58 10 (3)	17.84 ± 2.75 18 (2)	19.21 ± 2.55 19 (2)	< 0.001*	< 0.001*	< 0.001*	< 0.001*	< 0.001*
**Group 2**	10.90 ± 3.18 11 (5.75)	12.00 ± 3.63 11.5 (5.5)	12.25 ± 2.63 12 (3.5)	0.151				
**Group 3**	16.85 ± 3.22 16 (4)	-	-	-				
Social competence								
**Group 1**	18.53 ± 4.41 18 (6)	37.84 ± 5.28 39 (5)	42.53 ± 6.10 43 (6)	< 0.001*	< 0.001*	< 0.001*	< 0.001*	< 0.001*
**Group 2**	22.45 ± 4.29 23 (6.5)	24.95 ± 4.83 24 (5.5)	22.95 ± 7.23 22 (7.75)	0.272				
**Group 3**	37.60 ± 6.27 36 (11)	-	-					
Stress management								
**Group 1**	18.42 ± 5.32 18 (5)	38.32 ± 5.22 38 (5)	42.89 ± 5.99 44 (5)	< 0.001*	< 0.001*	< 0.001*	< 0.001*	< 0.001*
**Group 2**	21.80 ± 3.24 22 (4.75)	23.05 ± 4.30 23 (5)	21.40 ± 4.66 31 (6.75)	0.287				
**Group 3**	34.85 ± 4.79 34 (6.5)	-	-					
Adaptation								
**Group 1**	15.63 ± 3.40 15 (3)	31.58 ± 4.93 32 (8)	36.00 ± 4.37 37 (4)	< 0.001*	< 0.001*	< 0.001*	< 0.001*	< 0.001*
**Group 2**	18.35 ± 3.76 18.5 (4)	19.15 ± 3.66 18 (6)	17.70 ± 5.17 17 (6)	0.364				
**Group 3**	30.10 ± 6.09 30 (12.5)	-	-					
General mood								
**Group 1**	20.95 ± 4.72 20 (4)	43.16 ± 6.07 44 (3)	48.05 ± 6.38 50 (5)	< 0.001*	< 0.001*	< 0.001*	< 0.001*	< 0.001*
**Group 2**	26.20 ± 4.36 26.5 (5)	28.55 ± 5.32 28 (6.5)	28.50 ± 8.64 25 (9)	0.222				
**Group 3**	43.45 ± 5.62 43 (10.5)	-	-					
Positive impression								
**Group 1**	9.95 ± 3.26 10 (3)	19.00 ± 3.38 19 (3)	21.58 ± 3.31 23 (2)	< 0.001*	< 0.001*	< 0.001*	< 0.001*	< 0.001*
**Group 2**	11.95 ± 2.04 12 (3)	12.40 ± 2.33 12 (2.75)	12.45 ± 3.49 12 (2)	0.565				
**Group 3**	17.65 ± 2.54 18 (1.75)	-	-					
Total of Emotional Intelligence								
**Group 1**	92.47 ± 20.96 92 (20)	187.74 ± 25.54 188 (23)	210.26 ± 27.65 217 (19)	< 0.001*	< 0.001*	< 0.001*	< 0.001*	< 0.001*
**Group 2**	111.65 ± 16.75 113 (22.5)	120.10 ± 16.98 117.5 (23)	115.25 ± 28.31 107.5 (25)	0.283				
**Group 3**	180.50 ± 22.27 178 (37)	-	-					

The Kruskal-Wallis test, The Friedman test, the Mann-Whitney U test were used * Statistically significant difference (*p* < 0.05),

Group1: opioid addicts who have emotion intelligence program, group 2: opioid addicts who did not have emotion intelligence program, group 3: Healthy control group

**Table 6:** Addiction Severity Index (ASI) among cases groups before intervention, and 12 weeks after intervention

Variables	Group 1 (*n* = 19)			Group 2(*n* = 20)			*p*-value of baseline time of 2 groups	*p*-value of 3rd follow-up time of 2 groups
	Baseline time (T1)	3rd follow-up (T3)	*p*-value of 2 measures	Baseline time (T1)	3rd follow-up (T3)	*p*-value of 2 measures		
	*N* (%)	*N* (%)		*N* (%)	*N* (%)			
**Medical status**								
No problems	11(57.9)	11(57.9)	0.158	15(75.0)	8(40.0)	0.025*	0.166	0.264
Mild to moderate	5(26.3)	8(42.1)		5(25.0)	12(60.0)			
Severe problems	3(15.8)	0(0.0)		0(0.0)	0(0.0)			
**Employment status**								
No problems	0(0.0)	1(5.3)	< 0.001*	9(45.0)	2(10.0)	0.040*	< 0.001*	0.167
Mild to moderate	5(26.3)	18(94.7)		10(50.0)	15(75.0)			
Severe problems	14(73.7)	0(0.0)		1(5.0)	3(15.0)			
**Alcohol abuse**								
No problems	17(89.5)	18(**94.7)**	0.547	20(100.0)	19(95.0)	0.311	0.136	0.970
Mild to moderate	2(10.5)	1(5.3)		0(0.0)	1(5.0)			
Severe problems	0(0.0)	0(0.0)		0(0.0)	0(0.0)			
**Drug abuse**								
No problems	1(5.3)	7(36.8)	< 0.001*	0(0.0)	0(0.0)	0.011*	0.008*	0.001*
Mild to moderate	4(21.1)	9(47.4)		14(70.0)	6(30.0)			
Severe problems	14(73.7)	3(15.8)		6(30.0)	14(70.0)			
**Legal status**								
No problems	16(84.2)	17(89.5)	0.307	18(90.0)	15(75.0)	0.379	0.297	0.413
Mild to moderate	1(5.3)	2(10.5)		2(10.0)	4(20.0)			
Severe problems	2(10.5)	0(0.0)		0(0.0)	1(5.0)			
**Family and social status**								
No problems	2(10.5)	3(15.8)	< 0.001*	8(40.0)	0(0.0)	0.003*	< 0.001*	0.010*
Mild to moderate	4(21.1)	16(84.2)		11(55.0)	14(70.0)			
Severe problems	13(68.4)	0(0.0)		1(5.0)	6(30.0)			
**Psychiatric status**								
No problems	1(5.3)	4(21.1)	< 0.001*	11(55.0)	1(5.0)	0.002*	< 0.001*	0.233
Mild to moderate	2(10.5)	14(73.7)		7(35.0)	16(80.0)			
Severe problems	16(84.2)	1(5.3)		2(10.0)	3(15.0)			

Chi square test * Statistically significant difference (*p* < 0.05)

Group1: opioid addicts who have emotion intelligence program, group 2: opioid addicts who did not have emotion intelligence program

**Table 7:** Multivariate regression between difference of total emotional intelligence (before program and 3months follow up) and other variables

Variables	B	Std. Error	Beta	t	*P* value	95.0% Confidence Interval	
						Lower Bound	Upper Bound
Have the EI program	87.799	20.374	0.685	4.309	0.0001*	46.347	129.250
**Personality disorder**							
Total PID	1.162	1.188	0.080	-0.978	0.335	-1.255	3.579
**Addiction Severity index scale**							
Drug abuse	-0.566	2.889	-0.018	-0.196	0.846	-6.444	5.312
Psychiatric status	0.510	2.605	0.025	0.196	0.846	-4.790	5.811
**AWARE score**	-0.457	0.330	-0.200	-1.387	0.175	-1.127	0.213

* Statistically significant difference (*p* < 0.05), PID: Personality inventory for DSM-5, AWARE: Advance Warning of Relapse

## Data Availability

No datasets were generated or analysed during the current study.

## Abbreviations

EI: Emotional Intelligence
HIV: The human immunodeficiency viruses
ASI: Addiction Severity Index
PID-5: Personality Inventory for DSM-5
SCL-90-Revised: The Symptom ChickList-90-Revised
AWARE: Advance Warning of Relapse
DSM-5: The Diagnostic and Statistical Manual of Mental Disorders, Fifth Edition
EIS: The Emotional Intelligence Scale
